# THE ALZHEIMER’S ASSOCIATION NATIONAL HELPLINE: EFFECTS ON FAMILY CAREGIVERS

**DOI:** 10.1093/geroni/igz038.795

**Published:** 2019-11-08

**Authors:** Nancy Hodgson, Darina V Petrovsky, Beth Kallmyer, Joanne Pike, Sam Fazio

**Affiliations:** 1 University of Pennsylvania, Philadelphia, Pennsylvania, United States; 2 Alzheimer’s Association Home Office, Chicago, Illinois, United States

## Abstract

Family caregivers of persons with dementia experience high rates of social isolation and limited access to emotional support. The Alzheimer’s Association National Helpline is an accessible and free resource available 24 hours/day, 365 days/year in which master’s-level clinicians offer confidential emotional support and information on resources in the form of “action steps.” We evaluated the preliminary effectiveness of the Helpline for family caregivers of persons with dementia. Between January and October 2018, 185 non-crisis, caregiver calls to the Helpline were assessed at the time of call, one week, and 1 month post-call for effects on caregivers’ self-reported emotional distress, ability to manage anxiety, implement plan of action and access of services. The mean age of callers was 56 years; 22% were non white; 79% were women. Callers reported significantly (p <.05) improved caregiver emotional distress (27% net improvement) and ability to manage anxiety (29% net improvement). At one week, 70% of callers had put action steps in place, and by 1 month 80% of callers had put action steps into place. Over 80% of callers reported action steps were “helpful” and 65% reported that they had accessed additional dementia support services. This study suggests that support provided via the Alzheimer’s Association National Helpline is effective at reducing caregiver emotional distress and improving the ability of callers to “take action”. The results provide support for a larger study investigating caller characteristics and core content of the calls that provided sustained benefit to standardize the key elements of Helpline calls.

